# Effectiveness of Onion Extract Gel on Surgical Scars in Asians

**DOI:** 10.1155/2012/212945

**Published:** 2012-08-08

**Authors:** Kumutnart Chanprapaph, Somsak Tanrattanakorn, Penpun Wattanakrai, Pranee Wongkitisophon, Vasanop Vachiramon

**Affiliations:** Division of Dermatology, Faculty of Medicine, Ramathibodi Hospital, Mahidol University, Rajthevi, Bangkok 10400, Thailand

## Abstract

*Background*. Onion extracts have been shown in vitro to accelerate wound healing. Results from clinical studies on surgical scars in Caucasians were disappointing. The aim of this study is to evaluate the effectiveness of onion extract gel in improving the cosmetic and symptoms of surgical scars in Asians. *Patients/Methods*. Twenty Asians who had new Pfannenstiel's cesarean section scars were recruited in this prospective double-blinded, split-scar study. Each side was randomly assigned treatment with onion extract gel or placebo at 7 days after surgery. The product was applied three times daily for 12 weeks. Subjects were evaluated at baseline and 4th and 12th weeks. Scar redness was assessed by calorimeter, scar height and pliability were assessed by blinded investigators, and scar symptoms and overall cosmetic improvement were assessed by subjects. *Results*. Sixteen subjects completed the study. A statistically significant difference between two sides of scar in terms of scar height and scar symptoms was found. There was no statistically significant difference in scar redness, scar pliability, and overall cosmetic appearance between two sides. *Conclusions*. The early use of topical 12% onion extract gel on Pfannenstiel's cesarean section scar in Asians resulted in the improvement of scar height and scar symptoms.

## 1. Introduction 

Wound healing, a complex and dynamic interactive process, is divided into 3 overlapping phases: inflammation, tissue formation, and tissue remodeling [1]. Cutaneous scars from surgical wound can result in normal asymptomatic scars to cosmetically unacceptable scars [2]. Scars are not only a cosmetic concern but they can also cause pain, itching, discomfort, contracture, and other functional impairment [3, 4]. Several well-proven interventions are available for scar treatment including intralesional steroid injection, surgical excision, cryotherapy, radiotherapy, dermabrasion, pulse dye, and carbon dioxide laser therapy [5, 6]. These treatments have variable success and require multiple therapeutic sessions. Therefore, prevention and early recognition of hypertrophic scars and keloids are essential in their management. 

Among preventive treatments available, onion extract-based topical gel has been marketed as a product to improve the appearance and texture of surgical scars [6]. Data from in vitro studies suggested that the onion extract exhibits anti-inflammatory, antiproliferative, bacteriostatic, and collagen downregulatory properties by its effect on fibroblast and mast cell [7–9]. Despite encouraging laboratory data, clinical studies of onion extract for scar treatment have been unsatisfactory [10]. However, the numbers of studies are sparse and have several limitations such as the study design and variations in scars based on anatomical site. Moreover, because these studies were performed in advanced-age Caucasians, the risks of hypertrophic scar or keloid development were relatively low. Therefore, we conduct a prospective, randomized, double-blinded, split-scar study to determine the effect of topical onion extract gel on the appearance and symptoms of newly developed Pfannenstiel's cesarean section scar compared to placebo in postpartum Asian women. We anticipated to strengthen the research methodology by limiting the variables stated above.

## 2. Materials and Methods

### 2.1. Study Population

The study was conducted at the Division of Dermatology, Ramathibodi Hospital, Mahidol University, Bangkok, Thailand, with the approval from of the university Institutional Review Board (IRB). The research protocol conformed to the guidelines of the Helsinki Declaration, and informed consent was obtained from subjects prior to enrollment.

Woman who underwent cesarean section with Pfannenstiel's incision were recruited from the Department of Obstetric and Gynecology. Eligible subjects were ones that have undergone cesarean section for the first time using absorbable suture materials and were able to follow study protocol. They were excluded if they had history of onion allergy or developed surgical complications.

### 2.2. Study Design

The study was designed as a double-blinded, randomized, split-scar study. Using a randomization table, each side of scar was randomly assigned into receiving either a vehicle-based gel or an onion extract gel containing 12% *Allium cepa* (Erasé gel, ABCA Pharma Lab Co., Ltd., Nonthaburi, Thailand). Each scar was divided into 3 parts: left, middle, and right. To minimize the effect of medication diffusion, 1 cm in the middle was left untreated. Both agents were identical and contained in opaque tubes that were labeled “right” or “left.” Each gel was applied on the corresponding side of the scar three times daily for 12 weeks starting on postoperative day 7. 

### 2.3. Assessment of Response

The outcomes were assessed by both objective and subjective evaluation at baseline and 4th and 12th week after starting the treatment. Photographic documentation was performed from the front side of the scar under the same conditions at baseline and each subsequent visit.

### 2.4. Objective Evaluations

For objective evaluation, colorimeter (ChromaMeter CR-231, Minolta Corporation, Osaka, Japan)was used to assess scar redness. We used the Commission Internationale de l' Eclairage L*a*b* color system, which allowed a color to be quantified on three axes: white-black (L*), red-green (a*), and yellow-blue (b*). We used the a* parameter with values from +60 to −60 (green) to measure subject's skin redness index (SRI) [11]. SRI from both sides of the scar was measured for 3 times and was calculated the mean.

### 2.5. Subjective Evaluations

Physician and subject evaluation were performed at baseline and on each subsequent visit. Two blinded investigators rated the scars independently on each side using 2 parameters: pliability and height. Grading of pliability ranged from 0 to 5: 0: normal pliability; 1: supple, flexible with minimal resistance; 2: yielding (giving way to pressure, offering moderate resistance but did not behave as a solid mass of scar); 3: firm (solid, inflexible unit, not easily moved, resistant to manual pressure); 4: banding (rope-like tissue that blanched with extension of scar, did not limit range of motion(ROM)); 5: contracture (permanent shortening of the scar producing deformity or distortion, limited ROM). Scar height was graded as 0 (flat), 1(<2 mm), 2 (2 to 5 mm), and 3(>5 mm). All participants scored the visual analogue scale ranging from 0 (absent) to 3(severe) to evaluate each side for pain, itching, discomfort, tightness, and hardness. This was added to give a total summation of 15. Subjects were also asked to rate the overall cosmetic improvement of each side of the scar using scores −1 (worse), 0 (no improvement), 1 (minimal improvement), 2 (moderate improvement), and 3 (marked improvement).

### 2.6. Safety Assessments

Any adverse effects that occurred after applying the products were recorded at each visit. 

### 2.7. Statistical Analyses

Statistical analysis was performed by using computer software (STATA/SE version 11.2, STATA Corp, College Station, TX). Categorical variables (i.e., gender, Fitzpatrick's skin type, and gravidity) were expressed as percentages and continuous variables (i.e., age, scar redness, scar pliability, scar height, scar symptoms, and overall cosmetic appearance) were expressed as mean. To compare the variables between the control group and the treatment group, a paired *t*-test was used. A *P* value of <0.05 was considered statistically significant. 

## 3. Results

### 3.1. Patient Demographics

**Figure 1:**
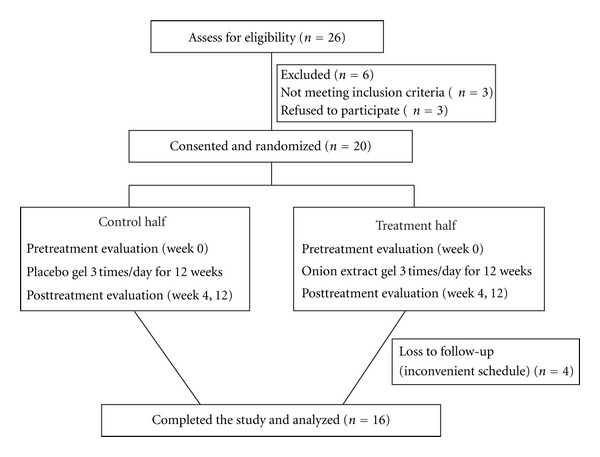
The flow chart showing number of eligible, refused to participate, recruited, randomized, and completed subjects.

Of the 26 subjects who were assessed for eligibility, 6 were excluded (3 declined to participate and 3 did not meet the inclusion criteria). Twenty female participants who met the eligibility criteria were enrolled. There were sixteen subjects who completed the protocol. Four subjects did not complete the study due to incomplete follow-up (Figure 1). Data from patients who failed to complete the study was excluded from the statistical analysis.

All of the participants were Thai women with Fitzpatrick's skin types varying from type 3 to 5, the majority were skin type 4 (75%). Subjects' age ranged from 19 to 43 years, with the median age of 31 years. Nine subjects (56.25%) were nulliparous and 7 subjects (43.75%) were multiparous (Table 1).

### 3.2. Calorimeter Measurement of Scar Redness

Seven subjects (43.75%) had a lower SRI on the onion extract side compared to control side at the 4th week and 5 subjects (31.25%) had a lower SRI on the onion extract side at the 12th week. The mean SRI decreased for both control and treatment sides at weeks 4 and 12. On the control side, the mean SRI changed from 10.65 ± 1.39 at baseline to 9.49 ± 1.32 and 8.90 ± 1.39 at week 4 and 12, respectively. The mean SRI of the treatment side was 10.67 ± 1.46 at baselines and 9.56 ± 1.24 and 9.03 ± 1.36 at the 4th and 12th week follow-up, respectively. There were no statistically significant differences in mean SRI between the control and treatment sides at any point in our evaluation.

### 3.3. Physician Evaluation: Scar Height and Pliability

**Figure 2:**
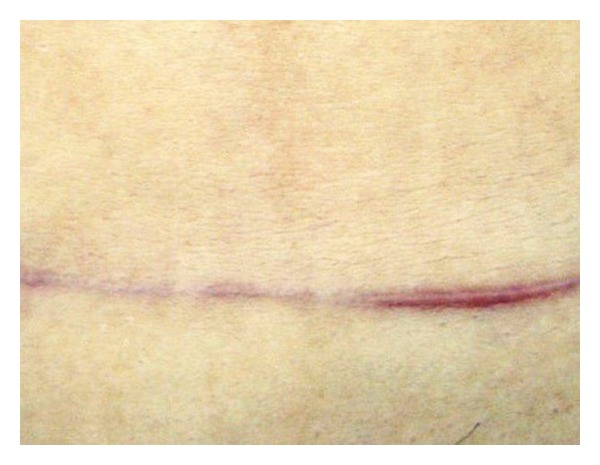
Photograph of a 32-year-old female subject at the 12th week follow-up. Onion extract and placebo were applied on the right and left side, respectively. Hypertrophic scar was visibly more elevated in the left side.

**Figure 3:**
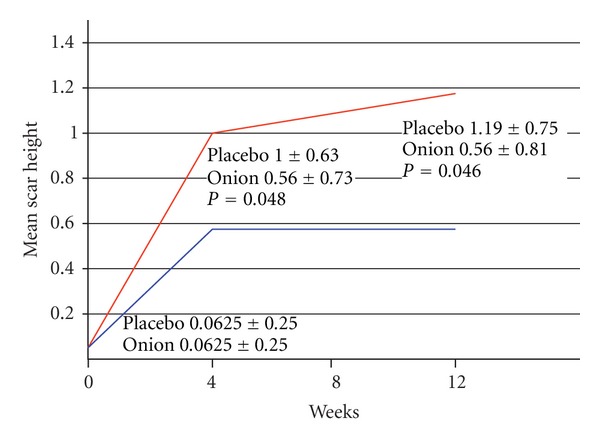
This figure demonstrates mean scar height on the placebo compared to the treatment side. A statistically significant difference favoring the treatment side was found at the 4th and 12th week follow-up. Red and blue line represents placebo and treatment side, respectively.

There was no difference in mean scar height between the control side and the treatment side at baseline. Six patients (37.50%) had a lower scar height on the onion extract side compared to the control side at the 4th week and 9 subjects (56.25%) had a lower scar height on the treatment side at the 12th week. A statistically significant difference in mean scar height between the two sides was found at the 4th week (1.00 ± 0.63 versus 0.56 ± 0.73, *P* = 0.048) and 12th week follow-up (1.19 ± 0.75 versus 0.56 ± 0.81, *P* = 0.046) (Figures 2 and 3).

There was no difference in mean scar pliability score between the two sides at baseline. Three patients (18.75%) had a lower scar pliability score on the onion extract side compared to the control side at the 4th week and 7 subjects (43.75%) had a lower scar pliability score on the onion extract side at the 12th week. However, this was not statistically significant (4th week: 0.56 ± 1.03 versus 0.25 ± 0.68, *P* = 0.10; 12th week: 1.31 ± 1.01 versus 0.75 ± 0.93, *P* = 0.06).

### 3.4. Subject Evaluation: Scar Symptoms and Overall Cosmetic Improvement

**Figure 4:**
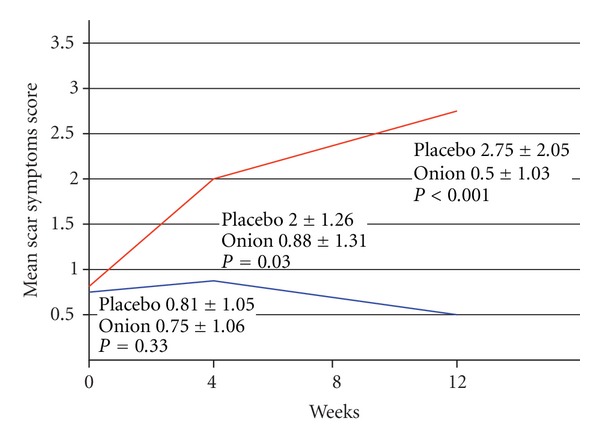
This figure compares mean scar symptoms score on the placebo and the treatment side. A statistically significant difference was found at the 4th and 12th week follow-up. Red and blue line represents placebo and treatment side, respectively.

At baseline, there was no difference in mean scar symptoms between the control side and the treatment side (0.81 ± 1.05 versus 0.75 ± 1.06). Ten subjects (62.50%) had a lower scar symptoms score on the onion extract side compared to the control side at the 4th week and 13 participants (81.25%) had a lower scar symptoms score on the onion extract side at the 12th week. A statistically significant difference in mean scar symptoms between the two sides was found at the 4th week (2 ± 1.26 versus 0.88 ± 1.31, *P* = 0.03) and 12th week follow-up (2.75 ± 2.05 versus 0.50 ± 1.03, *P* < 0.001) (Figure 4).

At the 4th week, all 16 control sides and 15 of 16 treatment sides were rated as no improvement (overall cosmetic improvement score = 0). One of 16 treatment sides was rated as worse (overall cosmetic improvement score = −1). At the 12th week, 15 of 16 control sides and 14 of 16 treatment sides were rated as no improvement; 1 of 16 control sides and 1 of 16 treatment sides were rated as minimal improvement (overall cosmetic improvement score = +1); 1 of 16 treatment sides was rated as moderate improvement (overall cosmetic improvement score = +2). There was no statistically significant difference in overall cosmetic improvement score at any point of evaluation.

### 3.5. Adverse Effects

Overall, subjects tolerated both onion extract and placebo gel well. No adverse effect was observed.

## 4. Discussion

It is difficult to predict scar development following surgical wound. It can vary from fine asymptomatic scars to hypertrophic scars and keloids leading to pruritus, pain, disfigurement, and psychological stress [1, 3, 4]. Hypertrophic scarring occurs within 4 to 8 weeks following wound closure, has a rapid growth phase for up to 6 months, and usually regresses over a period of a few years. The incidence of hypertrophic scar varies from 40% to 70% [6]. Keloid formation is found in all races. However, dark-skinned individuals have higher susceptibility to develop keloid, with an incidence of 5 to 15 times higher in African Americans and 3 to 5 times higher in Asians compared to Caucasians [3, 12]. The risk of thickened scar after surgery is also higher in certain parts of the body: shoulder and scapular area, anterior chest, lower abdomen, earlobe, and any region overlying a bony prominent [13]. 

Various treatment options exist for treating hypertrophic scars and keloids [4], most of which require multiple visits and have limited success. Therefore, prevention of problematic scars is undoubtedly more effective than treatment. Identifying high-risk individuals, minimizing skin tension and inflammatory response after surgery by using appropriate suture materials, and ascertaining clean surgery and good wound care are simple prophylactic measures. Different strategies are available for scar prevention; these include vitamin E-based remedies, pressure therapy, silicone gel and sheeting and topical onion extract [6]. Among these remedies, an extract of *Allium cepa*, or onion, is one of the top selling products [2]. It has been claimed by its manufacturers to improve the appearance and texture of surgical scars [1, 14]. Quercetin, the active ingredient, has been shown in in vitro studies to decrease fibroblast proliferation, inflammation, extracellular matrix deposition, and stabilized mast cells [7, 9, 15, 16]. Despite the encouraging laboratory results, documented studies and clinical reviews have revealed that onion extract neither improves nor prevents hypertrophic scars [2, 10, 17, 18]. However, these studies had small sample sizes and were conducted using merely subjective mode of measurement. Scars developed in these studies varied in anatomical site and the cause of injury. Subjects were elderly Caucasians, those with low risk to develop scars. We expected to strengthen the research methodology by controlling as many variables as possible.

In this study, we selected split-scar design to have optimal internal control. Pfannenstiel's cesarean section scar was chosen instead of midline scar to control the anatomical variation, knowing that the right and left side of this transverse incision should be more or less similar. Hence, the scar comparison in this study had matching location in the same patient with the identical cutaneous injury. Furthermore, the internal control design of this study eliminated confounding factor of differences in suture material, diverse surgical technique, and experience of surgeon. In addition, all of our subjects were Asian women with skin type 3 to 5. By choosing a group inherently of high risk of developing hypertrophic scars, we hoped to enhance a significant result.

The result of our study showed a significant difference in mean scar height and mean scar symptoms between control side and the treatment side at weeks 4 and 12. Although there were no statistically significant difference in mean scar pliability scores at any point of evaluation, a nearly significant difference was found at the 12th week (*P* = 0.06). In addition, there was tendency towards a better outcome with 43.7% of subjects showing lower pliability score on the treatment side at the 12th week. However, our study did not demonstrate any benefit of onion-extract-based topical gel in terms of scar redness and overall cosmetic improvement score between the control group and treatment group at the 4th and 12th week follow-up. 

Jackson and Shelton [10] evaluated the effectiveness of topical onion extract gel in improving the appearance and symptoms of postsurgical scars compared to topical emollient in 17 subjects who had undergone Mohs surgery. Subjects were divided into 2 groups: onion extract group and emollient group. Each group applied a designated topical product 3 times a day for 1 month. No statistically significant difference was found between pre- and posttreatment evaluations of scar erythema and pruritus in patients using topical onion extract gel. However, limitations included the study design, small sample size, and short treatment duration and follow-up period.

According to a randomized, double-blinded, split-scar study by Chung et al. [17], 24 subjects with new surgical wounds were treated with either onion extract gel or petrolatum ointment. There was no statistically significant difference in terms of scar symptoms, erythema, hypertrophy, and overall cosmetic improvement between the onion-extract-treated side and the petrolatum-treated side. However, all subjects were Caucasians who had undergone Mohs or excisional surgery for skin cancer and the average age was 65 years. Despite receiving any treatment, the nature of trauma and the subject's demographic would have contributed to an excellent scarring outcome. Participants in our study had higher risk of developing scars (e.g., younger age, darker skin type, and scar located at lower abdomen). Therefore, the reduction in scar height and symptoms in our study was highly reliable.

In general, all subjects tolerated the products well without any reports of adverse effects. Unlike the study by Jackson and Shelton, irritation was reported in 33.33% of subjects in the onion extract group [10]. 

The limitations in this study were the small sample size and the lack of long-term follow-up, since keloids may not develop until years after the event [3, 4]. Further studies, incorporating a larger number of subjects and a longer follow-up period up to a year are essential to provide additional information. 

## 5. Conclusion

The use of 12% onion extract gel on cesarean section scars three times daily during early postoperative period reduced height and scar symptoms without side effects. There was tendency towards improvement of scar pliability. Scar redness and overall cosmetic results did not improve. It can be used as adjunctive treatment to prevent hypertrophic scar from surgical wounds.

## Figures and Tables

**Table 1:** Demographic data of the subjects.

Demographic characteristic	Number of subjects (%)
Gender	
Female	16 (100%)
Age	
19–23 years	1 (6.25%)
24–28 years	3 (18.75%)
29–33 years	6 (37.50%)
34–38 years	4 (25%)
39–43 years	2 (12.50%)
Gravida	
Nulliparous	9 (56.25%)
Multiparous	7 (43.75%)
Fitzpatrick's skin type	
Type 3	1 (6.25%)
Type 4	12 (75%)
Type 5	3 (18.75%)
